# Promiscuous Speciation with Gene Flow in Silverside Fish Genus *Odontesthes* (Atheriniformes, Atherinopsidae) from South Western Atlantic Ocean Basins

**DOI:** 10.1371/journal.pone.0104659

**Published:** 2014-08-15

**Authors:** Graciela García, Néstor Ríos, Verónica Gutiérrez, Jorge Guerra Varela, Carmen Bouza Fernández, Belén Gómez Pardo, Paulino Martínez Portela

**Affiliations:** 1 Sección Genética Evolutiva, Facultad de Ciencias, UdelaR, Montevideo, Uruguay; 2 Departamento de Genética, Facultad de Veterinaria, Campus de Lugo, Universidad de Santiago de Compostela, Lugo, Spain; Institute of Biochemistry and Biology, Germany

## Abstract

The present paper integrates phylogenetic and population genetics analyses based on mitochondrial and nuclear molecular markers in silversides, genus *Odontesthes*, from a non-sampled area in the SW Atlantic Ocean to address species discrimination and to define Managements Units for sustainable conservation. All phylogenetic analyses based on the COI mitochondrial gene were consistent to support the monophyly of the genus *Odontesthes* and to include *O. argentinensis*, *O. perugiae-humensis* and some *O. bonariensis* haplotypes in a basal polytomy conforming a major derivative clade. Microsatellites data revealed somewhat higher genetic variability values in the *O. argentinensis-perugia* populations than in *O. bonariensis* and *O. perugia-humensis* taxa. Contrasting population genetics structuring emerged from mitochondrial and microsatellites analyses in these taxa. Whereas mitochondrial data supported two major groups (*O. argentinensis-perugia-humensis vs. O. bonariensis*-*perugiae-humensis* populations), microsatellite data detected three major genetic entities represented by *O. bonariensis*, *O. perugiae-humensis* and an admixture of populations belonging to *O. argentinensis-perugiae* respectively. Therefore, the star COI polytomy in the tree topology involving these taxa could be interpreted by several hypothetic scenarios such as the existence of shared ancestral polymorphisms, incomplete lineage sorting in a radiating speciation process and/or reticulation events. Present findings support that promiscuous and recent contact between incipient species sharing asymmetric gene flow exchanges, blurs taxa boundaries yielding complicated taxonomy and Management Units delimitation in silverside genus *Odontesthes* from SW Atlantic Ocean basins.

## Introduction

The New World presents multiple examples of atherinid species flocks or adaptive radiations arising from habitat transitions [1], [2], [3]. Silverside fish from South America constitute a exciting model to understand the scenario of fish speciation driven by divergent natural selection [1], [2].

The silverside genus *Odontesthes* includes 20 nominal species [4] distributed in marine, estuarine and freshwater environments of tropical and temperate regions in South America [5]. Most *Odontesthes* species co-occur in the same habitats and they are characterized by a great morphological homogeneity [6]. The low morphological divergence between species and the high meristic plasticity within species together with the tendency of local populations to form micro-geographic habitat associations had led to complicated taxonomy among silverside taxa [7].

Among freshwater representative species, two of them *O. bonariensis* and *O. hatcheri* are endemic of rivers and lakes located east of the Andes in subtropical and temperate areas [8]. The distribution of these species was originally allopatric: *O. hatcheri* occuring in the South (Patagonia), whereas *O. bonariensis* occupying Central and Northern Argentina, South Brazil and Paraguay. The occurrence of the spontaneous hybridization between both species in a communal laboratory tank has been reported [9].

On the other hand, marine silversides generally have similar life history strategies, occurring in large numbers in semi-isolated populations in estuaries and coastal lagoons [10], [11], [12], [1]. Ten species of *Odontesthes* are endemic of a chain of small shallow lakes spread along the South Western Atlantic Ocean coastal plain [13], [14]. Among them, in Patos Lagoon estuary and its adjacent marine coastal area occurs *O. argentinensis* and *O. incisa*, whereas in the freshwater habitats of Patos-Mirim lagoon system can be found *O. bonariensis, O. humensis*, *O. retropinnis and O. aff. perugiae*.

Most *Odontesthes* species represent economically important resources for artisanal and recreational fisheries in South America and particularly *O. bonariensis* shows a great potential for aquaculture development [15].

The identification of incipient ecological species represents an opportunity to investigate the current evolutionary process where adaptive divergence and reproductive isolation are associated [2]. Beheregaray and Sunnucks [2] found that niche divergence due to estuarine colonization by marine silverside fish led to isolation by adaptation and speciation in the presence of high gene flow, one of the most convincing reports of parapatric speciation in aquatic organisms from the Southern Hemisphere. Beheregaray et al. [16] explored the role of adaptive diversification and recent sea-level changes as evolutionary drivers in the *O. perugiae* species complex which comprises several allopatric and sympatric morphotypes found in the lakes and rivers of southern Brazil, Uruguay and northern Argentina [17]. Most morphotypes have uncertain taxonomic status and are endemic to the vast system of lakes of the Coastal Plain of Rio Grande do Sul State (CPRS), southern Brazil [17]. Beheregaray et al. [16] performed a phylogeographic reconstruction of radiations in the South American coastal freshwater *O. perugiae* species complex, and also reported some of the most rapid speciation rates for a vertebrate group.

Gene flow among hybridizing species with incomplete reproductive barriers blurs species boundaries, while selection under heterogeneous local ecological conditions or along strong gradients may counteract this tendency [18]. Thus, phylogeographic approach provides a valuable framework to identify signatures of divergent natural selection associated with ecological divergence and the possible occurrence of reticulation events among incomplete reproductively isolated taxa.

In this study we implement a phylogeographic analysis based on mtDNA coding sequences (cytochrome oxidase subunit I, COI) and ten microsatellite loci to access in the species boundaries and to test possible reticulation and introgression events among *Odontesthes* taxa from the SW Atlantic Ocean, the Río de la Plata estuary and in the Uruguay River basins. At the same time this information will contribute for a long-term success of Management Units for sustainable conservation of these taxa in fisheries and aquaculture.

## Materials and Methods

### Sample collection and DNA extraction

All sampling protocols for this scientific study were approved by CNEA (Comisión Nacional de Experimentación Animal) from Uruguay.

**Figure 1:**
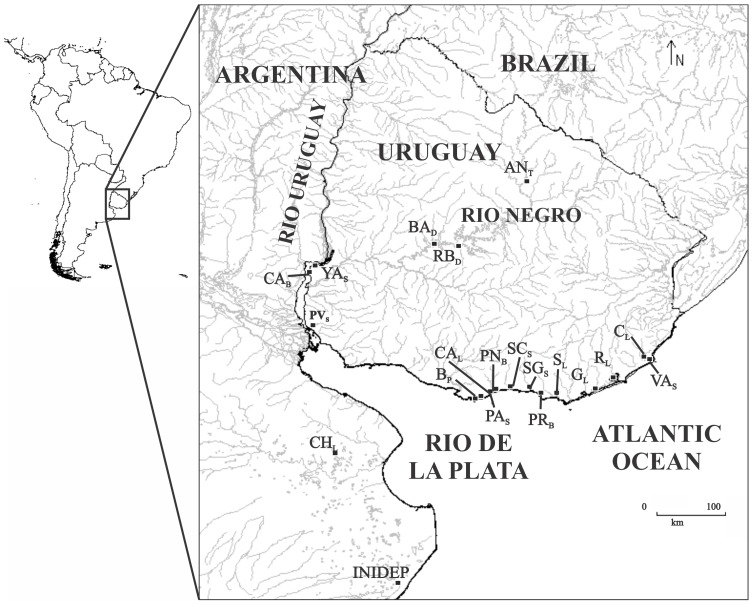
Distribution map of 20 sampling sites through three major areas, lower Uruguay and Negro river (UNR) basins, the Río de la Plata (RP) estuary, and associated coastal lagoons and sites from SWAtlantic Ocean (AC) in *Odontesthes* as follows: UNR- Las Cañas beach (CA_B_), Yaguarete stream (YA_S_), Pavón stream (PV_S_), Baygorria dam (BA_D_), Rincón del Bonete dam (RB_D_), Ansina town (AN_T_); RP- Buceo Port (B_P_), Hatchery and Carrasco lake (CA_L_), Pando stream (PA_S_), Pinar beach (PN_B_), Solis Chico stream (SC_S_); Solis Grande stream (SG_S_); Piriapolis beach (PR_B_), Sauce Lagoon (S_L_), Chascomus Lagoon (CH_L_), Argentina; AC-, Garzón Lagoon (G_L_), Rocha Lagoon (R_L_), Castillos Lagoon (C_L_), Valizas stream (VA_S_), National Institute for Fisheries Research and Development (INIDEP), Argentina.

A total of 163 individuals of *Odontesthes* from 20 sampling sites through three major regions, the Río de la Plata (RP) estuary (N = 45), Lower Uruguay and Negro river (UNR) basins (N = 23) and Atlantic coast (AC) sites of SWA Ocean (N = 95) were included in the present study. All these samples were primarily ascribed to *O. bonariensis* (*Ob*), *O. perugiae* species complex (*Op*), *O. humensis* (*Oh*), *O. argentinensis* (*Oa*) and only two specimens from *O. incisa* (*Oi*) according to Dyer [19] morphological diagnosis. Tissue samples were obtained from artisanal gillnets fisheries operating in these areas during 2006–2012. The sampled areas are shown in Figure 1 and Appendix S1. Sample codes are as follows: collecting site name and the corresponding environments in lowercase (i.e.: S =  stream, P =  port, B =  beach, L =  lagoon or lake, D = dam, T =  town). Tissues of the voucher specimens were deposited in the collection of the Evolutionary Genetics Section in the Faculty of Sciences, University of the Republic, Montevideo, Uruguay.

Genomic DNA of sacrificed specimens was isolated from muscle tissue (fixed in ethanol 95%) using sodium chloride protein precipitation, followed by ethanol precipitation modified from Medrano et al. [20].

### PCR amplifications and sequencing of the mitochondrial COI gene

A fragment of 650 bp from the COI gene was amplified using FishF2 and FishR1 primers [21]. Reaction volume was 10 µL containing 10X supplied buffer, 0.25 mM MgCl_2_, 0.2 mM of each dNTP (10 mM), 0.25 µM of each primer (10 µM), 0.1 units of *Taq* DNA polymerase (Invitrogen) and approximately 100 ng/ul of template DNA. Cycling conditions consisted of one initial denaturation at 94°C for 5 min followed by 35 cycles of 94°C for 30 s, 52°C for 30 s, 72°C for 1 min and a final extension of 72°C for 10 min.

Amplified COI products were sequenced directly on both strands in a Perkin-Elmer ABI Prism 377 Automated Sequencer (MACROGEN, Seoul, Korea). Sequence alignments were performed using Clustal X 1.8 [22].

### Statistical analyses of sequences from COI data set

Corrected estimates of pairwise sequence divergence were obtained using Kimura's [23] two-parameter algorithm (K2P) implemented in MEGA 5.0 [24]. Within a population, DNA polymorphism was measured by calculating the proportion of segregating sites (S), the haplotype diversity (h) [25], and the nucleotide diversity (π) [25] with ARLEQUIN v3.11 [26] and DnaSP version 4.50 [27] programs. Tajima's [28] and Fu's [29] tests implemented in DnaSP 4.50 [27] were performed to check the mutation/drift equilibrium and any departure from neutrality. Significance of Fu's *Fs* [29] and Tajima's *D* [28] values was evaluated using the coalescent algorithm comparing the observed value with a null distribution generated by 10,000 replicates, and giving an empirical population sample size and the observed number of segregating sites.

### Phylogenetic analysis and divergence time estimates of the mitochondrial gene

The phylogeographic relationships among mitochondrial COI haplotypes in *Odontesthes* populations from the sampled area were assessed by using two different methodologies. A non-model based method (MP, maximum-parsimony) was implemented in PAUP* 4.0b10 [30] following an equally weighted MP analysis using heuristic search (MULPARS option, stepwise addition, tree-bisection-reconnection [TBR] branch swapping, 100 replicates). A strict consensus between rival trees was computed to reconcile equally parsimonious topologies. The degree of confidence assigned to nodes in the trees was assessed by bootstrapping with 500 replicates.

On the other hand, two model based approaches were also used, i.e., maximum-likelihood (ML) and Bayesian inference (BI), implemented in PAUP* 4.0b10 [30] and BEAST v.1.5.4 [31], respectively.

In ML and BI analyses, the best-fitted nucleotide substitution model for each data set was determined in Modeltest v.3.7 [32] based on the Akaike information criterion [33], which simultaneously compares multiple nested or non-nested models. In the COI data set among the 56 models of nucleotide substitution, the best fit was the HKY+Γ model [34] with gamma distribution (Γ). The gamma distribution shape parameter value was 0.18. The likelihood scores estimated for these models were used as the prior settings for the ML analysis in the data set (−lnL = −1602.50). Heuristic search (again with 100 replicates of stepwise addition and TBR branch swapping) in ML analyses was implemented in PAUP* 4.0b10 [30]. The robustness of the nodes was determined after 1,000 bootstrapping replicates as implemented in PhyML 3.0 (http://atgc.lirmm.fr/phyml), according to the algorithm developed by Guindon et al. [35]. In this case, the NNI (a fast nearest neighbour edge interchange search) swapping algorithm option was implemented. Nonparametric bootstrap values above 75% were considered to be robust support for clades [36].

All trees were rooted by means of an outgroup criterion using sequences of *O. regia*, *O. incisa*, *O. smitti*, *O. hatchery* and *O. platensis* and a more distantly taxon *Atherina hepsetus* retrieved from the GenBank.

For the data set, divergence time of nodes and the age of the most recent common ancestor (tMRCA) were estimated with the BEAST v.1.5.4 software [31]. This program performs Bayesian statistical inferences of parameters by using MCMC (Monte Carlo Markov chain) as a framework. Input files were generated with Beauti v.1.5.4 [31] assuming uncorrelated lognormal trees and a Yule speciation process as prior information. The nucleotide substitution model and its parameter values were selected according to the Modeltest v.3.7 [32] results. An uncorrelated lognormal relaxed molecular clock, which allows rate variation among lineages, was implemented using an estimated rate for mitochondrial genome of 0.023 [2]. We carried out two independent runs of 10 million generations. Trees and parameters were sampled every 1,000 iterations, with a burn in of 10%. Results of each run were visualized in the Tracer v.1.5 program [37] to ensure that stationarity has been achieved and that convergence has been reached. Each analysis was repeated many times to optimize the operators of parameters until no suggestion message appeared in the log file. The timing of clade divergence and the tMRCA were estimated in million years ago (Mya) with a mean and a 95% HPD (lower and upper). Posterior probabilities and the maximum credibility tree were calculated using the TreeAnnotator v.1.5.4 software [31].

### AMOVA, isolation by distance and historical demography

To determine the genetic structure of *Odontesthes* samples the variance components among hierarchical partitions in the dataset were assessed by Analysis of Molecular Variance (AMOVA) [38]. The Euclidean metric of Excoffier et al. [38] was used to construct the pairwise distances matrix. The genetic variation was partitioned into three components, i.e., among groups (Ф_CT_), among populations within groups (Ф_SC_), and among individuals within populations (Ф_ST_), after disregarding either their original populations or their groups. For both molecular markers, populations were ascribed to three major sampling areas such as Atlantic coast (AC), Rio de la Plata (RP) and Uruguay and Negro river basins (UNR), and different grouping hypotheses for the populations were tested. The significance of the observed Ф-statistics was tested using the null distribution generated from 3,000 non-parametric random permutations of the data matrix variables and *P*-values were adjusted with sequential Bonferroni corrections for multiple comparisons [39].

Relationships and geographical distribution of the haplotypes were analysed in the haplotype network constructed with NETWORK v. 4.6.0.0 (http://www.fluxus-engineering.com/sharenet.htm), which implements the median-joining method, in the absence of recombination [40]. The network was optimized using maximum parsimony criterion.

Population subdivision and the level of genetic isolation among sampling sites were measured assuming an infinite sites model [41]. Pairwise estimates Ф-statistics were calculated in ARLEQUIN v3.11 [26].

To determine to what extent the geographic distance could explain the genetic differentiation among locations, a test for isolation by distance was performed using the Mantel test [42]. In this case, this test determines if there is a significant correlation between the geographic distance matrix (represented by the minimum coastline or river contour distance in kilometers) and the pairwise Fst matrix between collecting sites. The significance of the Z value (Mantel coefficient) was calculated using random permutation procedures implemented in the Mantel Non-parametric Test Calculator 2.0 [43]. Statistical significance was accessed through 1,000 permutations.

To assess to the historical demography of *Odontesthes* we compared the observed frequency distribution of pairwise nucleotide differences among haplotypes (i.e., mismatch distribution) in relation to the expected under a sudden population expansion model [44] implemented in ARLEQUIN v3.11 [26] and DnaSP version 4.50 [27] programs. The significance of the assumed model was tested using the sum of squares deviations (SSD) between the observed and expected data by means a parametric bootstrapping approach (1,000 permutations) and considering the Harpending's raggedness index [45]. The mismatch distribution will be multimodal in stable populations and unimodal in expanding ones. The time of a possible population expansion (τ) can be calculated as τ = 2*u*t [44], where τ is the mode of the mismatch distribution and *u* is the mutation rate of the sequence (such that *u* = µm_T_, where µ is the mutation rate/site/generation and m_T_ is the number of nucleotide base pairs). If the sudden expansion model was not rejected, then τ was converted to time since expansion (*t*) in years before present as follows: [YBP (*t* = τ/2*u*)]. For *Odontesthes* silversides, the mtDNA substitution rate was estimated in 0.023 [2]. Because time (*t*) is measured in generations and the age at sexual maturity for *Odontesthes* was calculated as minimum population doubling time 1.4–4.4 years (http://www.fishbase.org), to convert to time since expansion in years, we have multiply by the generation time of a mean 2.9 years.

### Analysis of microsatellite markers

A total of 120 individuals from 13 populations were analyzed using these nuclear markers (Appendix S1). Ten polymorphic microsatellite loci developed for *Odontesthes* were amplified: Odon02, Odon09, Odon27, Odon38, Odon39 [2]; and Obo01, Obo26; Obo46; Obo54 and Obo77 [46]. The forward primer of each pair was fluorescently labeled as follows: Odon02, Odon25, Odon39, Obo01, Obo54 with 5′-FAM; Odon27, Odon38, Obo26 and Obo77 with 5′-HEX; and finally Odon09 with 5′-NED.

PCR amplifications were carried out in a reaction volume of 10 µl (final concentrations in parenthesis) each containing DNA extract (400 ng/ul); dNTPs (0.1 mM each); primers (10 µM each); MgCl2 Invitrogen (0.8–2.5 mM); Taq DNA Polymerase Invitrogen (0.04 U/µl); and Invitrogen buffer (1X). Amplification conditions were those proposed by [2] and [46] respectively. The PCR reactions were carried out in a Verity 96-Well Thermal Cycler (Applied Biosystems) and the PCR products separated on an ABI 377 automated sequencer. The amplified fragments were genotyped using an ABI 3730 DNA Sequencer (Applied Biosystems) and visualization of the results was performed using the program GeneMapper 3.7 software (Applied Biosystems). Alleles were scored using a GeneScan 500 LIZ Size Standard and Genotyper software (Applied Biosystems, Inc.).

### Statistical and population structure analyses based on nuclear markers

Among all populations, only 13 were analyzed with microsatellites. To implement the analysis of the *Odontesthes* data set, based on biogeographic criteria and to avoid statistical bias due to the low number of samples in some collecting sites, the populations were first ascribed to different taxa as follows: *Oa* including population from G_L_ collecting site; *Ob* belonging to populations from CA_L_, S_L_ and C_L_; *Oph* populations from CA_B_, BA_D_, RB_D_; finally *Oap* embracing populations from B_P_, PN_B_, PA_S_, SC_S_, SG_S_, PR_B_ and R_L_. The number of alleles, the allelic richness, the expected heterozygosity corrected for sampling bias, the observed heterozygosity, the polymorphic information content and the estimated null allele frequency were calculated for each locus in the whole population per taxon using CERVUS version 3.0.3 [47]. GENEPOP 4.0.10 [48] was used to perform the exact test for Hardy-Weinberg (HW) equilibrium by microsatellite loci (test multi-population) and by population (test multi-locus) using the Markov chain method with 1,000 iterations. Linkage disequilibrium between loci and deviations from Hardy-Weinberg equilibrium for each locus were tested by a Markov chain method following the algorithm of Guo and Thompson [49] and using the Bonferroni [50] correction for multiple comparisons (α = 0.05). All the analyses outlined above were implemented in GENEPOP 4.0.10 [48]. Wright's *F-statistics* (Fis, Fst, and Fit [51]) over populations and loci were calculated by FSTAT version 2.9.3.2 [52]. To detect the presence of scoring errors or the possible presence of null alleles, we analyzed the genotypic matrices obtained with the Micro-Checker software [53].

Neighbor Joining tree based on D_A_ distance [54] was constructed using Populations, 1.2.30 software package [55].

An analysis of population subdivision and clustering of individual genotypes was implemented with STRUCTURE v. 2.2 [56] by a MCMC method. We considered 1 to 13 different populations (K = 1 to K = 13). Ten independent runs employing an admixture model were implemented with a burn-in period length of 50,000 iterations, followed by 100,000 MCMC replicates. The average of these independent runs was calculated and the true value of “K” was accessed following the approach detailed in the manual of STRUCTUREv. 2.2 (http://pritch.bsd.uchicago.edu/structure.html).

Different groups of hypotheses and populations as sources of variation were assessed in the AMOVA considering all ten loci using ARLEQUIN 3.1 software package [26]. Furthermore, F_ST_ values for pairwise comparisons of the 13 *Odontesthes* populations and their significance level for genetic differentiation (P = 0.05) and Rst were tested additionally with FSTAT [52].

### Population divergence and migration rates from both molecular markers

To discriminate between the relative effects of divergence and gene flow on the speciation process, we analyzed our data set under the Isolation with Migration model [57]. The “isolation with migration” model in IMa does not assume gene flow and genetic drift are in equilibrium, making it the most appropriate for recently diverged populations that share haplotypes and alleles due to both gene flow and ancestral polymorphism. The model assumes that an ancestral population splits into two descendant populations that may continue to exchange genes after separation. Following [1] we consider *Ob* as a freshwater sister taxon of *Oa* and *Oph*, sharing a common freshwater ancestor with these taxa.

The method estimates posterior probability distributions for both ancestral and actual population sizes, directional migration rates between the two populations, and the time elapsed since population splitting. An MCMC approach is used to draw a sample from the posterior distribution of genealogies and to estimate three types of population parameters: population size (*θ* = 4*Nu*), splitting time (*t* = *Tu*, where *T* is the time in generations since the common ancestry, and it is of the same order of 4*N*) and migration rates (2*NM* = 4*Nu*×m/2). The priors were finally set as follows: the upper bound of population sizes *q* = 10, splitting times *t* = 4 and migration rates *m* = 2, respectively. We run the MCMC simulations with 100,000 burn-in steps and 10,000.000 sampled genealogies. The posterior distributions of migration rates and population sizes are derived analytically from the sampled genealogies.

## Results

### Genetic variation in the mitochondrial COI gene in Odontesthes species from SWA Ocean basins

This study includes a data set of 655 bp of mitochondrial COI gene from 156 individuals belonging to populations of *O. argentinensis, O. perugia, O. humensis* and *O. bonariensis* (GenBank accession numbers: KJ854753–KJ854894, see Appendix S1). Moreover, other sequences from *Odontesthes* species and one more distantly related genera (*Atherina hepsetus*) were retrieved from the GenBank and included for both the pairwise distance comparisons and the phylogenetic analyses.

Among 36 COI haplotypes initially assigned to *O. argentinensis*, 30% of them were shared with *O. perugiae* and 25% with *O. humensis* respectivelly. Among 7 COI haplotypes initially grouping *O. bonariensis* sequences, 58% of them were shared with *O. perugiae* and 8% with *O. humensis*. Therefore we partitioned the statistical analysis in two different data sets: *O. argentinensis-perugiae-humensis* (*Oaph*) and *O. bonariensis-perugiae-humensis* (*Obph*).

The *Oaph* populations showed higher haplotype diversity (*h*) and nucleotide diversity (π) than *Obph* (Table 1). Thirty six haplotypes were found in *Oaph* populations whereas only seven in *Obph* taxa. Except for the three most common haplotypes (H_1 and H_2 in *Obph* and H_6 in *Oaph*), most haplotypes represented rare variants that explained the observed haplotype diversity in each taxa (Appendix S1). A significant excess of low-frequency haplotypes and thereby negative and significant values of both Tajima's and Fu's neutrality tests were observed in *Oaph* indicating a departure from neutrality, whereas *Obph* presented only negative and significant values in Tajima's D test (Table 1). These values would be consistent with populations that experienced demographic expansion scenarios or alternatively selective sweeps.

**Table 1:** Estimates of DNA polymorphism in COI gene of *Odontesthes* populations from SW Atlantic Coast, Río de la Plata estuary and Uruguay-Negro River basins.

	Base pairs	Variable Sites	S	Number of Haplotypes	Haplotype Diversity	π	Kimura 2P Distance (Tv+Ts)	D	Fs
*Oaph*	684	40	36	36	0.843 (0.034)	0.003 (0.013)	0.007 (0.001)	−2.397 (P<0.01)	−54.046 (P<0.00)
*Obph*	684	20	10	7	0.696 (0.058)	0.002 (0.006)	0.004 (0.002)	−2.797 (P<0.001)	−0.715 (P>0.10)

S  =  Average of polymorphic segregating sites; Haplotype diversity (h  =  gene) (Nei, 1987); π  =  Nucleotide diversity (Nei, 1987). Corrected Kimura 2P distances (1980). D  =  Neutrality test (Tajima, 989). Fs  =  Neutrality test (Fu, 1997). Standard deviation in brackets (SD).

In *Oaph* populations the average of the corrected pairwise K2P sequence divergence between COI haplotypes was higher than in *Obph* (Table 1). The average pairwise distances between haplotypes of *O. argentinensis-perugiae-bonariensis-humensis* taxa and other *Odontesthes* species included in present study (*O. regia*, *O. platensis*, *O. smitti* and *O. hatchery*) was 0.047±0.011 (mean ± SE), whereas the divergence between the former and *O. incisa* was 0.075±0.018. The average divergence between the ingroup and the outgroup *A. hepsetus* was 0.611±0.153.

### Phylogenetic analyses

**Figure 2:**
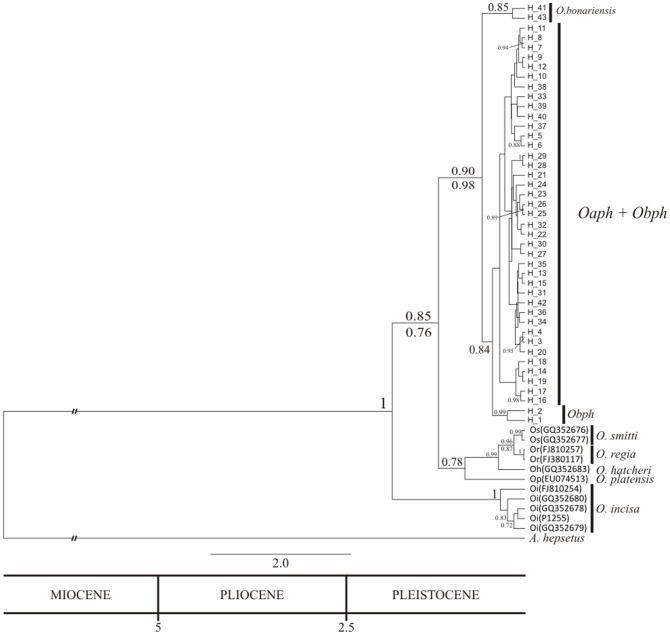
Tree topology generated using the HKY+Γ model of molecular evolution based on 43 COI gene haplotypes (H) of *Odontesthes* from lower Uruguay and Negro river basins, the Río de la Plata estuary, SWA Ocean basins. Bayesian phylogeographic inference framework implemented in beast 1.5.4 and the estimated divergence dates. Numbers above branches refer to the Bayesian posterior probability of occurrence for clades while bootstrap support values from ML bootstrap are shown below branches. The bottom bar summarizes the time-scale divergence dates in Mya.

Present phylogenetic analyses included 43 haplotypes from *Oaph* and *Obph* populations. All performed phylogenetic analyses (ML and beast) conducted using the HKY+Г model of sequence evolution, clearly identified a major monophyletic and recently derivate clade with a high posterior probability of occurrence, including minor monophyletic clades which collapsed in a basal polytomy joining most of the 41 haplotypes of *Oaph* and a well supported clade of *Obph* (Fig. 2). Other minor clade integrated by two *O. bonariensis* haplotypes collapsed basal to the major clade. Other species from the genus *Odontesthes* (*O. regia*, *O. smitti*, *O. hatchery* and *O. platensis*) were reciprocally monophyletic in relation to the minor clade of *O. bonariensis* and the major derivate clade, whereas *O. incisa* was the most basal taxon of the genus *Odontesthes*. Simultaneously we used a reference calibration time for nodes by assuming a substitution rate of conventional rate for mitochondrial genome of 0.023 mutations/site/per million years [16] to capture a plausible time interval (lower and upper estimate) for clade divergence. All *Odontesthes* clades showed a high probability to have diverged between 0.1 and 2.5 Mya (Quaternary). The divergence between the genus *Atherina* and *Odontesthes* occurred in the Miocene.

### Population genetic structure, isolation by distance and historical demography

Table 2 shows the pairwise F_ST_ values of the COI data set among the 15 collecting sites analyzed with this marker. Low population genetic structure was detected among localities ascribed to *Oap* from RP estuary and AC areas respectively. Nevertheless, these localities appeared divergent to those ascribed to *Obph* from some RP estuary sites, and UNR basins. Remarkably the CA_L_ collecting site seems to be the most divergent from all the remaining ones. The most plausible population structuring based on COI data set among tested hypotheses in the AMOVA was addressed following two different grouping criteria: (a) assigning all populations to two-group of samples; (b) forming three groups of populations (Table 3). The two-group hypothesis (a) pointed out that most genetic variation was distributed among groups (Φ_CT_), suggesting a remarkably higher level of genetic structure when samples from marine and estuarine morphs ascribed to *Oaph* populations was considered as a separate group from the other including freshwater samples from *Obph* taxa.

**Table 2:** Pairwise F_ST_ values based on COI data set of *Odontesthes* populations from SW Atlantic Coast, Río de la Plata estuary and Uruguay-Negro River basins.

	YA_S_ CA_B_ PV_S_	BA_D_ RB_D_ AN_T_	R_L_	SC_S_	SG_S_	PA_S_	PN_S_	G_L_	B_P_	C_L_ VA_S_	S_L_	CA_L_	PR_B_	CH_L_	INIDEP
YA_S__CA_B__PV_S_ (*Op*)	0														
BA_D__RB_D__AN_T_ (*Oph*)	0.036	0													
R_L_ (*Oap*)	**0.229**	0.085	0												
SC_S_ (*Oap*)	**0.261**	0.092	0.023	0											
SG_S_ (*Oap*)	**0.315**	0.103	−0.031	0.013	0										
PA_S_ (*Oap*)	0.191	0.076	−0.04	−0.043	−0.074	0									
PN_S_ (*Oa*p)	**0.147**	0.040	0.024	−0.042	0.049	−0.013	0								
G_L_ (*Oa*)	**0.249**	0.088	−0.026	0.022	−0.038	−0.035	0.036	0							
B_P_ (*Oap*)	**0.262**	0.086	−0.020	−0.056	−0.065	−0.057	0.009	−0.026	0						
C_L__VA_S_ (*Obp*)	0.206	**0.207**	**0.405**	**0.582**	**0.616**	**0.551**	**0.483**	**0.503**	**0.539**	0					
S_L_ (*Ob*)	0.133	0.086	**0.259**	**0.305**	**0.340**	**0.301**	**0.215**	**0.288**	**0.302**	**0.196**	0				
CA_L_ (*Ob*)	**0.480**	**0.313**	**0.312**	**0.400**	**0.450**	**0.387**	**0.283**	**0.355**	**0.377**	**0.709**	**0.220**	0			
PR_B_ (*Oap*)	**0.333**	0.195	**0.211**	**0.253**	**0.313**	**0.250**	**0.026**	**0.243**	**0.255**	**0.667**	**0.332**	**0.499**	0		
CH_L_ (*Ob*)	0.067	0.021	**0.238**	**0.273**	**0.318**	**0.270**	0.173	**0.260**	**0.272**	0.147	0.087	**0.450**	**0.333**	0	
INIDEP (*Oa*)	**0.195**	**0.112**	0.122	0.092	**0.189**	0.103	−0.051	0.141	0.141	**0.503**	**0.246**	**0.321**	0.100	0.214	0

F_ST_ significant values are in bold (*P* = 0.05). (See Fig. 1 and Appendix S1).

**Table 3:** Analysis of molecular variance (AMOVA) based on COI gene of *Odontesthes* populations from SW Atlantic Coast, Río de la Plata estuary and Uruguay-Negro River basins.

Hypothesis	Source of variation	*df*	Sum of squares	Variance components	Percentage of variation	Φ statistics
a	Among groups	1	8.864	0.10574 Va	21.21	Φ_CT_ = 0.21209
	Among population within groups	13	8.690	0.03416 Vb	6.85	Φ_SC_ = 0.08695
	Within populations	139	49.855	0.35867 Vc	71.94	Φ_ST_ = 0.28060
b	Among groups	2	9.588	0.09463 Va	19.44	Φ_CT_ = 0.19441
	Among population within groups	11	7.419	0.03316Vb	6.81	Φ_SC_ = 0.08456
	Within populations	138	49.539	0.35897 Vc	73.75	Φ_ST_ = 0.26253

Two grouping hypotheses among all tested: *a*) conforming two groups of populations as follows: 1-populations from S_L_, C_L_, CA_L_ and CH_L_ *vs.* 2-populations from VA_S_, R_L_, SC_S_, SG_S_, PA_S_, PN_B_, G_L_, B_P_, PR_B_, INIDEP, CA_B_, YA_S_, BA_D_, RB_D_ and PV_S_; *b*) separating three groups of samples as follows:1-populations from CA_B_, PV_S_, YA_S_, BA_D_ and RB_D_; 2-populations from VA_S_, R_L_, SC_S_, SG_S_, PA_S_, PN_B_, G_L_, B_P_, PR_B_, INIDEP and 3- populations from S_L_, C_L_, CA_L_ and CH_L_. (See Fig. 1 and Appendix S1).

**Figure 3:**
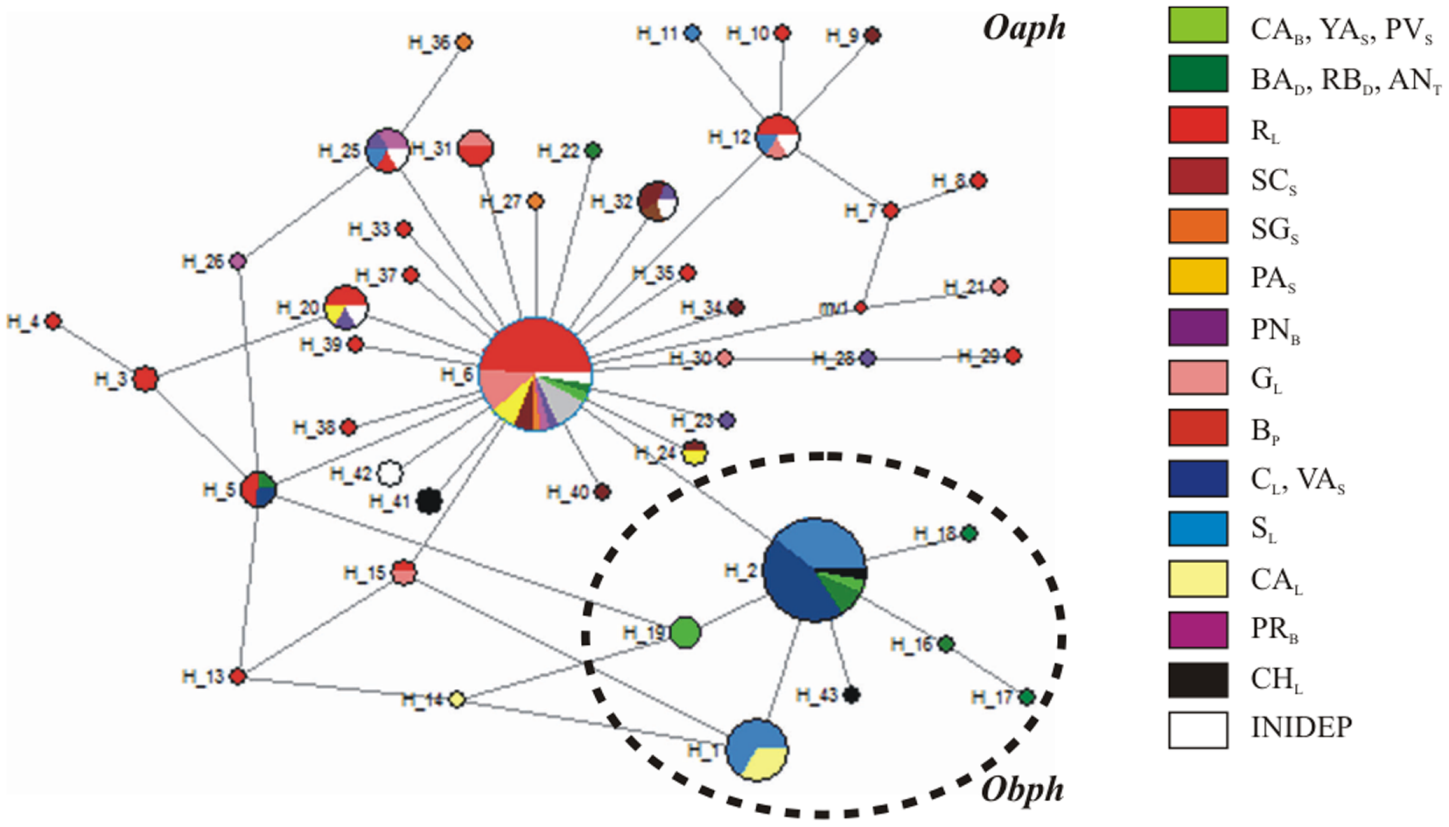
Haplotype network (constructed with NETWORK v. 4.6.0.0 software) of *Oaph* and *Obph* taxa. Black dots represent missing haplotypes and circle size is proportional to haplotype frequency. Different colours in each circle indicate the collecting sites as described in the Figure 1.

The haplotype network based on COI gene (Fig. 3) showed a strikingly star-shaped topology including the two most frequent haplotypes with a high proportion of singletons, typical of populations that have suffered a recent demographic expansion. One of the most frequent and central haplotypes (H_6) including samples of *Oaph* is present in 11 sampling sites and is shortly interconnected by one to three step-mutations to most haplotypes of the network, belonging to *Oaph* populations. A single step mutation separated H_6 from the other most frequent haplotype (H_2), which included samples belonging to *Obph* taxa from 6 sampling sites. Remarkably, the network topology showed some loops involving the central H_6 and H_2 haplotypes and their respective derivate ones. Therefore these alternative links may be representing equally good connections due to homoplasy or perhaps the existence of peripheral reticulation events among them.

Taking into account all collecting sites, negative values in Mantel test were observed (r = −0.126, p = 0.050), showing a negative correlation between genetic and geographic distances and excluding the isolation by distance model of population differentiation in *Odontesthes*.

**Figure 4:**
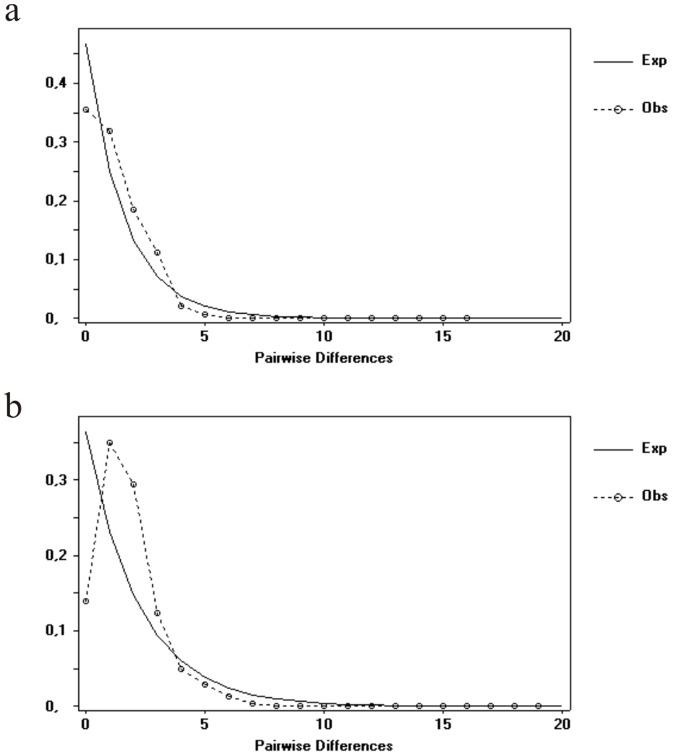
Mismatch distribution in *Odontesthes* species under the growth–decline population model using mtDNA COI data set. (a). *Oaph* and (b). *Obph* populations.

Figure 4 shows an unimodal mismatch distribution pattern in the COI data set which adjusted to the distribution predicted by the growth–decline population model [44] in the *Oaph* populations. The sum of squares deviations was SSD = 0.148 (P>0.06) and Harpending's Raggedness index was 0.486. The estimate parameter under the model was τ = 3.218. The time of expansion-decline in *Oaph* based on a substitution for this marker was estimated to have started around 227,000 YBP. In the *Obph* populations data set the sum of squares deviation value was SSD = 0.180 (P>0.05) and Harpending's Raggedness index was 0.492, therefore population growth-decline hypothesis was accepted for this taxon. The estimate parameter under the model was τ = 7.843 and the time of expansion-decline in *Obph* populations were estimated to have started around 497,000 YBP.

### Genetic variability in multi-locus nuclear data

The measures of microsatellite genetic variation including total number of alleles, allelic richness, heterozygosity and HWE deviation for each locus and the corresponding average across all loci per taxon are showed in Table 4. Significant departures from Hardy-Weinberg equilibrium were found at some loci of the following populations: *Oap* (Odon27 and Obo54); *Oph* (Obo26, Odon27, Odon39 and Odon02); *Ob* (Obo39, Obo54 and Odon02). This could be due to a Wahlund effect, with a reduction of heterozygosity caused by any subpopulation structure. The null hypothesis of equilibrium was accepted across all loci in *Oa* population and when a global test across loci and populations was performed (p<0.05, Table 4). Based on Fis estimates there was also no support for inbreeding at any locality, except from *Ob* populations (average across all loci 0.177).

**Table 4:** Estimates of DNA polymorphism based on ten microsatellite loci in *Odontesthes* populations from SW Atlantic Coast, Río de la Plata estuary and Uruguay-Negro River basins.

		*Oap*					*Oph*					*Ob*					*Oa*				
Locus	A_T_	A	r_A_	Ho	He	*P*	A	rA	Ho	He	*P*	A	rA	Ho	He	*P*	A	rA	Ho	He	*P*
Obo01	71	57	3.882	0,897	0,980	0,212	16	3.749	0,917	0,957	0,537	15	3.526	0,865	0,915	0,074	7	3.600	0,800	0,933	0,380
Obo26TUF	19	12	2.797	0,686	0,743	0,104	4	4.000	1,000	1,000	1,000	9	3.060	0,861	0,819	0,820	3	1.800	0,400	0,378	1,000
Obo46	34	30	3.093	0,810	0,800	0,420	13	3.706	0,778	0,948	0,029	11	2.963	0,811	0,788	0,572	7	3.172	0,833	0,833	0,543
Obo54TUF	41	25	3.200	0,740	0,836	0,009	13	3.770	0,778	0,961	0,081	13	3.154	0,833	0,834	0,039	6	3.467	1,000	0,911	1,000
Obo77TUF	31	23	3.541	0,941	0,916	0,773	16	3.922	1,000	0,987	1,000	6	2.507	0,657	0,682	0,717	3	2.600	1,000	0,733	1,000
Odont02	30	26	3.679	0,927	0,944	0,275	12	3.542	0,667	0,915	0,012	12	3.177	0,722	0,842	0,000	6	3.367	0,800	0,889	0,640
Odont09	20	16	3.291	0,927	0,864	0,931	8	3.111	0,667	0,824	0,289	10	2.890	0,829	0,769	0,876	7	3.600	1,000	0,933	1,000
Odont27	11	11	2.681	0,814	0,715	0,029	3	2.305	0,364	0,636	0,014	5	1.779	0,405	0,384	0,069	3	2.077	0,667	0,530	1,000
Odont38	42	36	3.761	0,966	0,958	0,975	14	3.686	0,917	0,946	0,287	11	3.120	0,838	0,832	0,103	8	3.564	0,833	0,924	0,084
Odont39	20	14	3.184	0,825	0,843	0,737	10	3.284	0,417	0,862	0,000	4	1.214	0,081	0,106	0,028	8	3.564	1,000	0,924	1,000

Total number of alleles (A_T_); number of alleles (A); allelic richness based on minimum sample size of 2 diploid individuals (r_A_); observed (H_O_) and expected (H_E_) heterozygosity; significant departure from Hardy-Weinberg equilibrium (P) (P<0.05).

Populations were ascribed to four taxa. *Oa* included only population from Garzon Lagoon (G_L_) collecting site. (See Fig. 1 and Appendix S1).

Micro-Checker analysis suggested heterozygote deficit at some loci, but not in all populations (p<0.05) and that the deficit of heterozygotes in these loci, were not due to stuttering or large allele dropout but it might be the result of null alleles, such as in the cases of Obo01 and Obo 54 in the *Oap* populations. The number of alleles per locus varied from 11 (Odon27) to 71 (Obo01) in the total sample. Average number of alleles ranged from 25 (*Oap*) to 5.8 (*Oa*) (Table 4). Allelic number was significantly lower in the locus Odon27 than the remaining in all populations. Allelic richness was significantly lower in the locus Odon27 for *Ob* populations than the remaining ones. The highest number of private alleles was detected in the *Oap* population using FSTAT package.

Exact test of genotypic linkage disequilibrium were not significant (p<0.05) after Bonferroni correction in the global approach, except for two pairs of loci (Odont 39-Odont27 and Obo01-Obo54).

### Population genetics structure based on microsatellites

Pairwise Fst values among 13 populations showed little differentiation for *Oap* taxa, as well as among those belonging to *Oph*, whereas the populations from *O. bonariensis* presented little to moderate genetic differentiation (Table 5). Moderate differentiation was evident between *Oap* and *Oph* populations whereas large differentiation (F_ST_>0.15) was evident between *Oph* and *Ob* taxa pairwise comparisons.

**Table 5:** Pairwise F_ST_ values based on ten microsatellite loci of *Odontesthes* populations from SW Atlantic Coast, Río de la Plata estuary and Uruguay-Negro River basins.

	YA_S_ CA_B_ PV_S_	BA_D_ RB_D_ AN_T_	R_L_	SC_S_	SG_S_	PA_S_	PN_S_	G_L_	B_P_	C_L_ VA_S_	S_L_	CA_L_	PR_B_
YA_S__CA_B__PV_S_ (*Op*)	0												
BA_D__RB_D__AN_T_ (*Oph*)	−0.019	0											
R_L_ (*Oap*)	**0.101**	**0.088**	0										
SC_S_ (*Oap*)	**0.116**	**0.102**	0.002	0									
SG_S_ (*Oap*)	**0.103**	**0.087**	0.007	**0.042**	0								
PA_S_ (*Oap*)	**0.114**	**0.102**	−0.012	0.003	0.001	0							
PN_S_ (*Oa*p)	**0.120**	**0.110**	−0.005	**0.024**	**0.021**	−0.006	0						
G_L_ (*Oa*)	0.118	**0.109**	−0.014	−0.004	0.014	−0.015	−0.001	0					
B_P_ (*Oap*)	**0.089**	**0.084**	0.002	0.018	0.011	0.001	0.014	0.008	0				
C_L__VA_S_ (*Obp*)	**0.261**	**0.237**	**0.176**	**0.212**	**0.213**	**0.202**	**0.207**	**0.192**	**0.156**	0			
S_L_ (*Ob*)	**0.308**	**0.269**	**0.192**	**0.227**	**0.233**	**0.229**	**0.219**	**0.205**	**0.191**	**0.08**	0		
CA_L_ (*Ob*)	0.257	**0.209**	**0.128**	**0.166**	**0.166**	**0.164**	**0.149**	**0.136**	**0.148**	**0.12**	0.018	0	
PR_B_ (*Oap*)	**0.084**	**0.079**	−0.004	−0.006	0.012	−0.007	0.003	0.004	0.006	**0.188**	**0.215**	**0.147**	0

F_ST_ significant values are in bold (*P* = 0.05). (See Fig. 1 and Appendix S1).

Among all grouping hypotheses tested, AMOVA results showed that three groups rendered the most plausible hypothesis with the largest percentage of variance within individuals, followed by among groups, whereas remaining components of variance value were very low (Table 6).

**Table 6:** Analysis of molecular variance (AMOVA) based on ten microsatellite loci in *Odontesthes* populations from Atlantic Coast, Río de la Plata estuary and Uruguay-Negro River basins.

Source of variation	*df*	Sum of squares	Variance components	Percentage of variation	Φ statistics
Among groups	2	67.258	0.515	15.68	Φ_CT_ = 0.15677
Among populations within groups	10	35.285	0.056	1.71	Φ_SC_ = 0.02025
Among individuals within populations	93	250.480	−0.019	−0.58	Φ_IS_ = −0.00697
Within individuals	106	289.500	2.731	83.19	Φ_IT_ = 0.16810

Three-groups hypothesis among all tested as follows: 1- populations from CA_B_, PA_S_, YA_S_, BA_D_ and RB_D_; 2- populations from R_L_, SC_S_, SG_S_, PA_S_, PN_B_, G_L_, B_P_, PR_B_ and 3- populations from S_L_, C_L_, CA_L_. (See Fig. 1 and Appendix S1).

**Figure 5:**
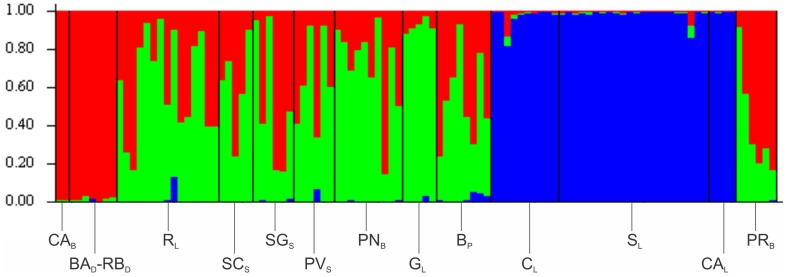
Estimated population structure based on the STRUCTURE analysis (K = 3) of the 10 nuclear microsatellite loci. Each bin or three colored vertical bar represents the estimated membership fraction of an individual into three major population clusters. Name of 13 collecting sites are above bars.

**Figure 6:**
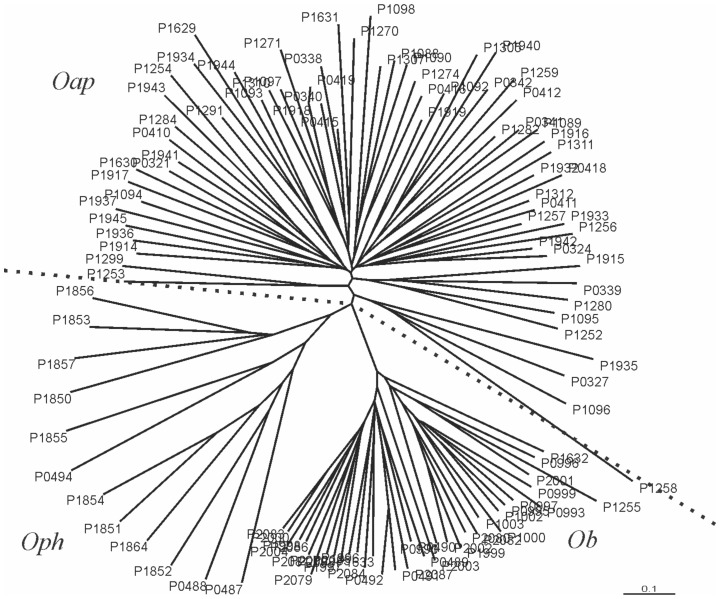
NJ tree based on a matrix of Nei's D_A_ genetic distance based on ten microsatellites data between *O. argentinensis, O.perugiae-humensis and O.bonariensis taxa* (calculated the Populations, 1.2.30, Langella 1999). Clusters indicate the distributions of individuals in relation to their corresponding taxon.

Both STRUCTURE (Fig. 5) and Neighbour-Joining (Fig. 6) analyses detected similar population genetic structure with the microsatellite data set. Using STRUCTURE, the posterior probability for K = 2 or K = 3 was very similar, but we assume the three cluster assignments Ln Pr (X|K = 3) = −5102, 89 as the most plausible one for our data set, whereas the probabilities for the other K values assayed were negligible (Fig. 5). Considering three clusters (K = 3) the individuals from S_L_, C_L_ and CA_L_ ascribed to *Ob* were assigned mainly to one genetically homogeneous cluster, whereas those from eight localities ascribed to *Oap* populations were mostly assigned separately, and finally three other localities (CA_B_, RB_D_ and BA_D_) were clustered as a separated and genetically homogeneous group belonging to *Oph* taxa (Figure 5).

The NJ tree based on Nei's genetic distance matrix detected the same three separated clusters belonging to the same aforementioned taxa (Figure 6).

### Isolation and Migration analyses

**Figure 7:**
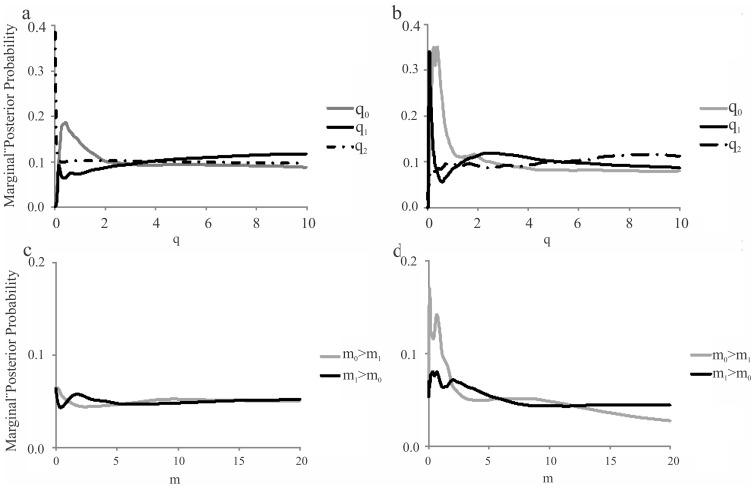
Marginal posterior probability distribution for the Isolation-with-migration demographic parameters obtained in IMa2 for both molecular markers. Curves are shown for estimates of effective population size in *Oaph* populations (q0), *Obph* bonariensis (q1) and ancestral population (q2) for COI data set (a) and for microsatellite data (b). Estimate migration rate in each pairwise comparison analyses of *Oaph* vs. *Obph* (m0>1) and *Obph vs. Oaph* (m1>0) for COI data set (c) and microsatellite data (d).

Because little to moderate genetic differentiation was detected in *Oaph* populations (based on both molecular markers) it was considered as single unit in the IMa2 under the two-population analyses (Fig. 7). Therefore, the performed analysis included the following two groups of populations: *Oaph* vs. *Obph*. The peaks of the six parameter estimate were confined to narrow ranges with the corresponding posterior distribution (Fig. 7). The estimate of population size for q0 (*Oaph*) under two markers was two-fold higher than that of the ancestral population (q2), indicating a possible population expansion events (Fig. 7a,b). Conversely, there seems not to be obvious change for the effective population size of q1 (*Obph*), whereas minor signals of bottleneck followed by population expansion appeared (Fig. 7a). Asymmetric migration rates were recovered from the IMa2 coalescent analysis (Fig. 7c,d) for both molecular markers. Microsatellite data (Fig. 7d) showed significant gene flow from *Oaph* to *Obph* whereas lower signal of gene flow was evident in the reverse direction. Conversely COI data (Fig. 7c) revealed higher posterior probability of retrospective gene flow from *Obph* to *Oaph* but not in the opposite direction.

## Discussion

### Phylogenetic analysis and Odontesthes species delimitation from SW Atlantic Ocean basins

All phylogenetic analyses based on the COI mitochondrial gene were consistent to support the monophyly of the genus *Odontesthes* and to include *Oaph* and some *Obph* haplotypes within a major derivate clade in an unresolved basal polytomy joining a minor clade integrated by other *O. bonariensis* sequences. The star polytomy included minor and poorly differentiated clades not geographically structured, with high posterior probability of occurrence and suggesting a recent radiation process. Furthermore, pairwise genetic distance values between these taxa were within the range of *Odontesthes* intraspecific levels. In contrast, phylogenetic analyses based on COI gene reported high posterior probability of occurrence in other cladogenetic events involving more distantly *Odontesthes* species. Therefore, *O. smitti*, *O. regia* and *O. hatcheri* integrated a monophyletic and sister group of the *O. bonariensis-argentinensis-perugiae-humensis* clade, whereas *O. incisa* split as a basal clade among the Atherinopsoidei.

Previous molecular phylogenetic analyses based on several mitochondrial genes proposed that *O. bonariensis* and *O. argentinensis* taxa comprise a common genetic lineage [1], [3]. It was consistent with their shared morphological characters [16] and with no statistically significant morphometric differentiation between them [58].

Remarkably in the present work, *Oph* populations were included in the aforementioned star polytomy together with *Oa* samples and some *Ob* haplotypes. Beheregaray and Sunnuck [2], based on demographic and phylogeographic history analyses of coastal *Odontesthes*, had proposed that *O. perugiae* species complex originated from an ancestral marine-estuarine lineage currently represented by *O. argentinensis*. At the same time, *O. argentinensis* would have emerged as the most recently derived marine species of the genus, having a common freshwater ancestor species with *O. bonariensis* having diversified during the Pleistocene [16]. Therefore, all these three taxa share common ancestry. Our results seem congruent with these findings, dating the aforementioned cladogenetic events about 0.1 and 2.5 Mya (since the Pleistocene-Post-Pleistocene).

Moreover, present analyses pointed out about the absence of phylogenetic signals to discriminate these four *Odontesthes* taxa which collapsed in the derivate basal polytomy. This could be interpreted as a hard polytomy and it would be explained by several hypothetic differentiation scenarios such as, the existence of shared ancestral polymorphisms, incomplete lineage sorting in radiating speciation process and/or reticulation events involving these taxa. Our results may be congruent with some recent events of hybridization since hybrid individuals tend to collapse in a basal polytomy when included in a cladistic analysis [59].

### Ancestral polymorphism, lineage sorting or introgression scenarios in the Odontesthes differentiation from SWA ocean basins?

Microsatellites data set revealed relatively higher heterozigosity values in the *Oaph* and *Oa* from G_L_ populations than in *Ob* taxon. These values were similar to those reported for *Oa* from southern Brazil [1], [16], but the heterozygosity values in the *Oph* species complex found by Behegaray et al. [2] were lower than in the present work.

Contrasting population genetics structuring emerged from mitochondrial and microsatellites analyses. AMOVA, Bayesian STRUCTURE inference and distance analyses based on microsatellite data yielded congruent assignment of each individual into three clusters (K = 3). Two of them including genetically homogeneous groups such as *Ob* from S_L_, C_L_ and CA_L_, and on the other hand, samples of *Oph* from two RB_D_, BA_D_ and CA_B_ (Fig. 6). The nuclear homogeneity detected in *Ob* should be expected since following Dyer [19], this taxon has its origins in lakes and lagoons of the Province of Buenos Aires, Argentina, connected with the Rio de la Plata estuary, and in Rio Grande do Sul, Brazil. Nevertheless, there are no records of *Ob* being native to Uruguay, and multiple deliberated introduction events from Argentina lagoons to different freshwater and estuarine environments for extensive aquaculture purposes have been reported in the former country [60].

On the other hand, a second cluster of individuals belonging to the remaining collecting sites represent a highly heterogeneous group of mixed ancestry between the marine-estuarine taxon *Oa* and the freshwater *Oph* taxa. This group showed a gradual variation in its nuclear genomic structure from the hypothetical shared ancestors. The variation is yet evident in the same marine-estuarine environment (i.e. R_L_, PN_B_ and B_P_) irrespective of its geographic distance and excluding the isolation by distance model of differentiation.

In contrast to microsatellite data, the AMOVA based on mitochondrial COI gene straightforward supports the existence of two groups: one including *Obph* populations and other integrated by *Oaph* samples. Consistent with genetic distances, haplotype network showed a strikingly star-shaped topology including two most frequent haplotypes shortly interconnected by branch lengths of one step mutation, one of them ascribed to *Oaphs* and the other one belonging to *Obph* taxa.

Taking into account all present data, the existence of hybridization events could have occurred among these four highly related taxa in sympatric estuarine areas. In particular introgression from *Oaph* taxa seems to have occurred toward *O. bonariensis* matrilineal genome. Contrasting IMa2 results from both molecular markers were consistent with a hypothetic hybridization and introgression scenarios between these taxa.

Previously, it has been reported [61] natural hybridization between both taxa (*O. bonariensis* vs. *O. argentinensis*) in the Salada Grande Lagoon from Argentina.

In the area under study, Pleistocene and Post-Pleistocene marine transgression produced habitat modifications and fragmentations in particular in coastal and shelf regions from South America [62]. As a principal consequence of these events, several rivers and coastal lagoons system were periodically separated from the Atlantic Ocean by sand bars, generating associated estuarine environments along South America coast [63]. Bamber and Henderson [11] supported that physically variable environments, such as estuaries and coastal brackish lagoons, pre-adapt silverside populations to invade, colonize and rapidly speciate into vacant freshwater niches. Incipient lakes may provide special conditions for radiations because of their remote access and low or absent competition from endemic lineages specialized in particular resources.

Nevertheless there is absence of absolute geographical and physiological barriers to gene flow that separate these *Odontesthes* populations in the study area. The Río de la Plata represents a continuous estuarine environment linking marine (SWA Ocean) to freshwater populations of *Odontesthes* inhabiting the Uruguay-Negro River basin.

Remarkably, the nuclear genomic heterogeneity detected in the *Oaph* group of populations would be explained by alternatively shared ancestral polymorphism, incomplete lineage sorting in radiating taxa and/or past recent process of reticulation events. In this sense, Beheregaray et al. [2] mentioned that *O. argentinensis-perugiae* populations share common mitochondrial haplotypes perhaps representing the matrilineal ancestor of them. These authors hypothesized that a fundamental issue is whether the *O. perugiae* morphotypes endemic to coastal lakes are the outcome of an evolutionary radiation or merely reflect phenotypic plasticity within a single species.

### Historical demography and Managements Units in sustainable fisheries and conservation strategies

Mismatch distribution and neutrality tests based in the COI data set yielded to different historical demographic scenarios in which population expansion could be proposed for *Oaph* populations. We note that the inferred IMa2 parameter of the effective population size in *Oaph* is larger than the supposed common ancestor and the *Obph*. This scenario could be consistent with secondary contacts between incipient species showing different combinations of nearly interspecific hybridizations and recent mixing populations.

Nevertheless, historical demographic parameters in *Obph* suggest that these populations have undergone past recent of more or less dramatic bottlenecks and founder effect episodes of population reductions, perhaps associated to freshwater Pampean mainland lakes and lagoons environments changes since later Pleistocene.

Beheregaray and Sunnuck [2] proposed that ecological speciation and “divergence-with-gene-flow” adjust to *Odontesthes* model of speciation. Present findings pointed out that promiscuous and recent contact between incipient species sharing high level of asymmetric gene flow blurs species boundaries yielding to complicated taxonomy and species delimitation among silverside genus *Odontesthes*.

Based on present data we propose that *Oaph* populations from the SW Atlantic Ocean, RP and UNR basins could be considered a metapopulation system for fishery policies and conservation purposes.

Present nuclear and mitochondrial data alert us about the sustainability in native and captive populations of *O. bonariensis*, within the aquaculture programs, regarding the moderate to relatively low genetic diversity found in this taxon, despite potential past/present reticulation events among its highly related sister species.

## Supporting Information

Appendix S1 Catalog number, collecting sites and areas, GenBank accession numbers and haplotype of *Odontesthes* individuals from lower Uruguay and Negro river (UNR) basins, the Río de la Plata (RP) estuary, and associated coastal lagoons and sites from SW Atlantic Ocean (AC). Samples included in the microsatellite population analyses are indicated with an X.
(DOC)
