# Supplementary material for: Promiscuous Speciation with Gene Flow in Silverside Fish Genus Odontesthes (Atheriniformes, Atherinopsidae) from South Western Atlantic Ocean Basins
Source: PLoS One. 2014 Aug 15;9(8):e104659. doi: 10.1371/journal.pone.0104659 (PMC4134232; doi:10.1371/journal.pone.0104659)
Supplement: Appendix S1 — Catalog number, collecting sites and areas, GenBank accession numbers and haplotype of Odontesthes individuals from lower Uruguay and Negro river (UNR) basins, the Río de la Plata (RP) estuary, and associated coastal lagoons and sites from SW Atlantic Ocean (AC). Samples included in the microsatellite population analyses are indicated with an X. (DOC) [file pone.0104659.s001.doc]

**Appendix S1**

| Specie | Catalog number | Area | Collecting site | GenBank Accession Number COI | COI Hap number | Microsatellites analyses |
| --- | --- | --- | --- | --- | --- | --- |
| *O. argentinensis* | P318 | AC | R_L_ | KJ854767 | 3 | X |
| *O. argentinensis* | P326 | AC | R_L_ | KJ854823 | 3 |  |
| *O. argentinensis* | P325 | AC | R_L_ | KJ854768 | 4 |  |
| *O. argentinensis* | P319 | AC | R_L_ | KJ854893 | 5 |  |
| *O. argentinensis* | P323 | AC | VA_S_ | KJ854891 | 5 |  |
| *O. argentinensis* | P324 | AC | VA_S_ | KJ854892 | 5 | X |
| *O. argentinensis* | P414 | AC | G_L_ | KJ854863 | 6 | X |
| *O. argentinensis* | P415 | AC | G_L_ | KJ854867 | 6 | X |
| *O. argentinensis* | P418 | AC | G_L_ | KJ854880 | 6 | X |
| *O. argentinensis* | P420 | AC | G_L_ | KJ854826 | 6 | X |
| *O. argentinensis* | P421 | AC | G_L_ | KJ854829 | 6 | X |
| *O. argentinensis* | P1269 | AC | R_L_ | KJ854860 | 6 |  |
| *O. argentinensis* | P1272 | AC | R_L_ | KJ854894 | 6 |  |
| *O. argentinensis* | P1274 | AC | R_L_ | KJ854868 | 6 | X |
| *O. argentinensis* | P1279 | AC | R_L_ | KJ854879 | 6 |  |
| *O. argentinensis* | P1281 | AC | R_L_ | KJ854856 | 6 |  |
| *O. argentinensis* | P1289 | AC | R_L_ | KJ854875 | 6 |  |
| *O. argentinensis* | P1290 | AC | R_L_ | KJ854872 | 6 |  |
| *O. argentinensis* | P1297 | AC | R_L_ | KJ854857 | 6 |  |
| *O. argentinensis* | P1298 | AC | R_L_ | KJ854869 | 6 |  |
| *O. argentinensis* | P1312 | AC | R_L_ | KJ854866 | 6 | X |
| *O. argentinensis* | P1315 | AC | R_L_ | KJ854859 | 6 |  |
| *O. argentinensis* | P1316 | AC | R_L_ | KJ854871 | 6 |  |
| *O. argentinensis* | P1317 | AC | R_L_ | KJ854877 | 6 |  |
| *O. argentinensis* | P1318 | AC | R_L_ | KJ854870 | 6 |  |
| *O. argentinensis* | P320 | AC | R_L_ | KJ854876 | 6 |  |
| *O. argentinensis* | P327 | AC | R_L_ | KJ854874 | 6 | X |
| *O. argentinensis* | P321 | AC | R_L_ | KJ854772 | 7 | X |
| *O. argentinensis* | P1305 | AC | R_L_ | KJ854773 | 8 | X |
| *O. argentinensis* | P1280 | AC | R_L_ | KJ854775 | 10 | X |
| *O. argentinensis* | P412 | AC | G_L_ | KJ854781 | 12 | X |
| *O. argentinensis* | P1273 | AC | R_L_ | KJ854779 | 12 |  |
| *O. argentinensis* | P1284 | AC | R_L_ | KJ854780 | 12 | X |
| *O. argentinensis* | P1309 | AC | R_L_ | KJ854778 | 12 |  |
| *O. argentinensis* | P322 | AC | R_L_ | KJ854782 | 13 |  |
| *O. argentinensis* | P423 | AC | G_L_ | KJ854791 | 15 |  |
| *O. argentinensis* | P1314 | AC | R_L_ | KJ854790 | 15 |  |
| *O. argentinensis* | P1307 | AC | R_L_ | KJ854822 | 20 | X |
| *O. argentinensis* | P1313 | AC | R_L_ | KJ854820 | 20 |  |
| *O. argentinensis* | P411 | AC | G_L_ | KJ854825 | 21 | X |
| *O. argentinensis* | P1291 | AC | R_L_ | KJ854886 | 25 | X |
| *O. argentinensis* | P1299 | AC | R_L_ | KJ854846 | 29 | X |
| *O. argentinensis* | P416 | AC | G_L_ | KJ854847 | 30 | X |
| *O. argentinensis* | P410 | AC | G_L_ | KJ854848 | 31 | X |
| *O. argentinensis* | P419 | AC | G_L_ | KJ854851 | 31 | X |
| *O. argentinensis* | P1270 | AC | R_L_ | KJ854849 | 31 | X |
| *O. argentinensis* | P1283 | AC | R_L_ | KJ854850 | 31 |  |
| *O. argentinensis* | P1271 | AC | R_L_ | KJ854881 | 33 | X |
| *O. argentinensis* | P1282 | AC | R_L_ | KJ854883 | 35 | X |
| *O. argentinensis* | P1311 | AC | R_L_ | KJ854887 | 37 | X |
| *O. argentinensis* | P1310 | AC | R_L_ | KJ854888 | 38 | X |
| *O. argentinensis* | P1308 | AC | R_L_ | KJ854889 | 39 |  |
| *O. argentinensis* |  | AC | INIDEP | EU074511 | 42 |  |
| *O. argentinensis* |  | AC | INIDEP | GQ352675 | 42 |  |
| *O. argentinensis* | P1629 | RP | B_P_ | KJ854858 | 6 | X |
| *O. argentinensis* | P1631 | RP | B_P_ | KJ854878 | 6 | X |
| *O. argentinensis* | P339 | RP | B_P_ | KJ854873 | 6 | X |
| *O. argentinensis* | P342 | RP | B_P_ | KJ854861 | 6 | X |
| *O. argentinensis* | P1089 | RP | PN_B_ | KJ854838 | 6 | X |
| *O. argentinensis* | P1253 | RP | PR_B_ | KJ854827 | 6 | X |
| *O. argentinensis* | P1256 | RP | PR_B_ | KJ854832 | 6 | X |
| *O. argentinensis* | P1916 | RP | SC_S_ | KJ854865 | 6 | X |
| *O. argentinensis* | P1919 | RP | SC_S_ | KJ854837 | 6 | X |
| *O. argentinensis* | P1932 | RP | SC_S_ | KJ854836 | 6 | X |
| *O. argentinensis* | P1933 | RP | SC_S_ | KJ854835 | 6 | X |
| *O. argentinensis* | P1934 | RP | SC_S_ | KJ854834 | 6 | X |
| *O. argentinensis* | P338 | RP | B_P_ | KJ854774 | 9 | X |
| *O. argentinensis* | P1088 | RP | PN_B_ | KJ854824 | 20 | X |
| *O. argentinensis* | P1086 | RP | PN_B_ | KJ854830 | 23 |  |
| *O. argentinensis* | P1917 | RP | SC_S_ | KJ854839 | 24 | X |
| *O. argentinensis* | P1091 | RP | PN_B_ | KJ854843 | 25 |  |
| *O. argentinensis* | P1252 | RP | PR_B_ | KJ854840 | 25 | X |
| *O. argentinensis* | P1254 | RP | PR_B_ | KJ854841 | 25 | X |
| *O. argentinensis* | P1257 | RP | PR_B_ | KJ854842 | 26 | X |
| *O. argentinensis* | P1937 | RP | SG_S_ | KJ854844 | 27 | X |
| *O. argentinensis* | P1090 | RP | PN_B_ | KJ854845 | 28 | X |
| *O. argentinensis* | P1630 | RP | B_P_ | KJ854855 | 32 | X |
| *O. argentinensis* | P1087 | RP | PN_B_ | KJ854852 | 32 |  |
| *O. argentinensis* | P1915 | RP | SC_S_ | KJ854854 | 32 | X |
| *O. argentinensis* | P1918 | RP | SC_S_ | KJ854853 | 32 | X |
| *O. argentinensis* | P340 | RP | B_P_ | KJ854882 | 34 | X |
| *O. argentinensis* | P1935 | RP | SG_S_ | KJ854885 | 36 | X |
| *O. argentinensis* | P341 | RP | B_P_ | KJ854890 | 40 | X |
| *O. argentinensis* | P1092 | RP | PN_B_ |  |  | X |
| *O. argentinensis* | P1093 | RP | PN_B_ |  |  | X |
| *O. argentinensis* | P1094 | RP | PN_B_ |  |  | X |
| *O. argentinensis* | P1095 | RP | PN_B_ |  |  | X |
| *O. argentinensis* | P1096 | RP | PN_B_ |  |  | X |
| *O. argentinensis* | P1097 | RP | PN_B_ |  |  | X |
| *O. argentinensis* | P1098 | RP | PN_B_ |  |  | X |
| *O. argentinensis* | P1255 | RP | PN_B_ |  |  | X |
| *O. argentinensis* | P1258 | RP | PN_B_ |  |  | X |
| *O. argentinensis* | P1914 | RP | SC_S_ |  |  | X |
| *O. argentinensis* | P1936 | RP | SG_S_ |  |  | X |
| *O. argentinensis* | P1941 | UNR | PA_S_ | KJ854862 | 6 | X |
| *O. argentinensis* | P1942 | UNR | PA_S_ | KJ854833 | 6 | X |
| *O. argentinensis* | P1943 | UNR | PA_S_ | KJ854831 | 6 | X |
| *O. argentinensis* | P1945 | UNR | PA_S_ | KJ854821 | 20 | X |
| *O. argentinensis* | P1944 | UNR | PA_S_ | KJ854864 | 24 | X |
| *O. argentinensis* | P1940 | UNR | PA_S_ |  |  | X |
| *O. bonariensis* | P2083 | AC | S_L_ | KJ854761 | 1 | X |
| *O. bonariensis* | P2084 | AC | S_L_ | KJ854762 | 1 | X |
| *O. bonariensis* | P2086 | AC | S_L_ | KJ854764 | 1 | X |
| *O. bonariensis* | P2090 | AC | S_L_ | KJ854753 | 1 | X |
| *O. bonariensis* | P1000 | AC | C_L_ | KJ854802 | 2 | X |
| *O. bonariensis* | P1002 | AC | C_L_ | KJ854810 | 2 | X |
| *O. bonariensis* | P1003 | AC | C_L_ | KJ854814 | 2 | X |
| *O. bonariensis* | P1004 | AC | C_L_ | KJ854815 | 2 |  |
| *O. bonariensis* | P1005 | AC | C_L_ | KJ854816 | 2 |  |
| *O. bonariensis* | P990 | AC | C_L_ | KJ854796 | 2 | X |
| *O. bonariensis* | P991 | AC | C_L_ | KJ854797 | 2 | X |
| *O. bonariensis* | P993 | AC | C_L_ | KJ854800 | 2 | X |
| *O. bonariensis* | P995 | AC | C_L_ | KJ854808 | 2 | X |
| *O. bonariensis* | P996 | AC | C_L_ | KJ854809 | 2 | X |
| *O. bonariensis* | P997 | AC | C_L_ | KJ854801 | 2 | X |
| *O. bonariensis* | P999 | AC | C_L_ | KJ854795 | 2 | X |
| *O. bonariensis* | P1632 | AC | S_L_ | KJ854784 | 2 |  |
| *O. bonariensis* | P1633 | AC | S_L_ | KJ854806 | 2 | X |
| *O. bonariensis* | P1634 | AC | S_L_ | KJ854807 | 2 |  |
| *O. bonariensis* | P1635 | AC | S_L_ | KJ854805 | 2 | X |
| *O. bonariensis* | P1636 | AC | S_L_ | KJ854804 | 2 |  |
| *O. bonariensis* | P1637 | AC | S_L_ | KJ854785 | 2 |  |
| *O. bonariensis* | P2003 | AC | S_L_ | KJ854755 | 2 | X |
| *O. bonariensis* | P2079 | AC | S_L_ | KJ854757 | 2 | X |
| *O. bonariensis* | P2080 | AC | S_L_ | KJ854758 | 2 | X |
| *O. bonariensis* | P2081 | AC | S_L_ | KJ854759 | 2 | X |
| *O. bonariensis* | P2082 | AC | S_L_ | KJ854760 | 2 | X |
| *O. bonariensis* | P2085 | AC | S_L_ | KJ854763 | 2 | X |
| *O. bonariensis* | P2088 | AC | S_L_ | KJ854765 | 2 | X |
| *O. bonariensis* | P1639 | AC | S_L_ | KJ854776 | 11 |  |
| *O. bonariensis* | P1640 | AC | S_L_ | KJ854777 | 12 |  |
| *O. bonariensis* | P1001 | AC | C_L_ | KJ854819 | 19 |  |
| *O. bonariensis* | P1641 | AC | S_L_ | KJ854884 | 25 |  |
| *O. bonariensis* | P1996 | AC | S_L_ |  |  | X |
| *O. bonariensis* | P1997 | AC | S_L_ |  |  | X |
| *O. bonariensis* | P1998 | AC | S_L_ |  |  | X |
| *O. bonariensis* | P1999 | AC | S_L_ |  |  | X |
| *O. bonariensis* | P2000 | AC | S_L_ |  |  | X |
| *O. bonariensis* | P2001 | AC | S_L_ | KJ854754 | 2 | X |
| *O. bonariensis* | P2002 | AC | S_L_ |  |  | X |
| *O. bonariensis* | P2004 | AC | S_L_ | KJ854756 | 2 | X |
| *O. bonariensis* | P2087 | AC | S_L_ |  |  | X |
| *O. bonariensis* | P2089 | AC | S_L_ |  |  | X |
| *O. bonariensis* | P490 | RP | CA_L_ | KJ854788 | 1 | X |
| *O. bonariensis* | P491 | RP | CA_L_ | KJ854787 | 1 | X |
| *O. bonariensis* | P492 | RP | CA_L_ | KJ854786 | 1 | X |
| *O. bonariensis* | P489 | RP | CA_L_ | KJ854783 | 14 | X |
| *O. bonariensis* |  | RP | CH_L_ | FJ810251 | 41 |  |
| *O. bonariensis* |  | RP | CH_L_ | GQ352682 | 41 |  |
| *O. bonariensis* |  | RP | CH_L_ | FJ810256 | 43 |  |
| *O. bonariensis* | P495 | UNR | CA_L_ | KJ854789 | 1 |  |
| *O. humensis* | P1854 | UNR | RB_D_ | KJ854811 | 2 | X |
| *O. humensis* | P1851 | UNR | RB_D_ | KJ854828 | 22 | X |
| *O. humensis* | P1852 | UNR | RB_D_ |  |  | X |
| *O. humensis* | P1855 | UNR | RB_D_ |  |  | X |
| *O. incisa* | P1255 | RP | PR_B_ | KJ854766 |  |  |
| *O. perugiae* | P1849 | AC | PV_S_ | KJ854817 | 19 |  |
| *O. perugiae* | P1864 | UNR | CA_B_ | KJ854812 | 2 | X |
| *O. perugiae* | P1856 | UNR | RB_D_ | KJ854803 | 2 | X |
| *O. perugiae* | P1857 | UNR | RB_D_ | KJ854798 | 2 | X |
| *O. perugiae* | P1865 | UNR | YA_S_ | KJ854813 | 2 |  |
| *O. perugiae* | P1860 | UNR | RB_D_ | KJ854769 | 5 |  |
| *O. perugiae* | P1853 | UNR | RB_D_ | KJ854770 | 6 | X |
| *O. perugiae* | P1859 | UNR | RB_D_ | KJ854771 | 6 |  |
| *O. perugiae* | P343 | UNR | AN_T_ | KJ854792 | 16 |  |
| *O. perugiae* | P1858 | UNR | RB_D_ | KJ854793 | 17 |  |
| *O. perugiae* | P1850 | UNR | CA_B_ | KJ854818 | 19 | X |
| *O. perugiae - O. humensis* | P488 | UNR | BA_D_ | KJ854794 | 2 | X |
| *O. perugiae - O. humensis* | P487 | UNR | BA_D_ | KJ854799 | 18 | X |
| *O. perugiae - O. humensis* | P494 | UNR | BA_D_ |  |  | X |

Collecting sites are described as follows: CA_B_- Las Cañas beach, YA_S_- Yaguarete stream, PV_S_-Pavón stream, BA_D_ -Baygorria dam, RB_D_- Rincón del Bonete dam, AN_T_- Ansina town; B_P_ - Buceo Port, CA_L_-Hatchery and Carrasco lake, PA_S_- Pando stream, PN_B_-Pinar beach, SC_S_-Solis Chico stream; SG_S_- Solis Grande stream; PR_B_- Piriapolis beach, CH_L_- Chascomus Lagoon, Argentina; S_L_ Sauce Lagoon, G_L_ Garzón Lagoon, R_L_ Rocha Lagoon, C_L_-Castillos Lagoon, VA_S_ Valizas stream, National Institute for Fisheries Research and Development (INIDEP), Argentina.
